# Susceptibility to Dental Caries and the Salivary Proline-Rich Proteins

**DOI:** 10.1155/2011/953412

**Published:** 2011-11-29

**Authors:** Martin Levine

**Affiliations:** Department of Biochemistry, University of Oklahoma Health Sciences Center, 940 S. L. Young Boulevard, Oklahoma City, OK 73104, USA

## Abstract

Early childhood caries affects 28% of children aged 2–6 in the US and is not decreasing. There is a well-recognized need to identify susceptible children at birth. Caries-free adults neutralize bacterial acids in dental biofilms better than adults with severe caries. Saliva contains acidic and basic proline-rich proteins (PRPs) which attach to oral streptococci. The PRPs are encoded within a small region of chromosome 12. An acidic PRP allele (Db) protects Caucasian children from caries but is more common in African Americans. Some basic PRP allelic phenotypes have a three-fold greater frequency in caries-free adults than in those with severe caries. Early childhood caries may associate with an absence of certain basic PRP alleles which bind oral streptococci, neutralize biofilm acids, and are in linkage disequilibrium with Db in Caucasians. The encoding of basic PRP alleles is updated and a new technology for genotyping them is described.

## 1. Introduction

Dental caries is the dissolution of the enamel and dentin in pits, fissures, and interdental regions of the teeth, eventually spreading to buccal and lingual surfaces. Since the release of “Oral Health in America: A Report of the Surgeon General” in May 2000, efforts have not advanced dental caries prevention, its risk of development, or its early detection [1]. Worse, the severity of caries has since been increasing in all socioeconomic groups [2]. Public preventive measures such as water fluoridation are not universally available, few rural populations have access to fluoridated water, and fluoridated dentifrices are only effective if the teeth are brushed regularly. Dental caries is the commonest chronic infectious disease of childhood in the United States affecting 28% of the population [3]. Childhood caries is a major reason for hospital visits [4], and it may destroy the deciduous dentition disproportionately in disadvantaged ethnic and socioeconomic groups [5]. A better means of identifying and protecting these children needs to be developed [6], but a simple method of identifying such individuals *a priori* at birth has proved elusive [7].

**Figure 1:**
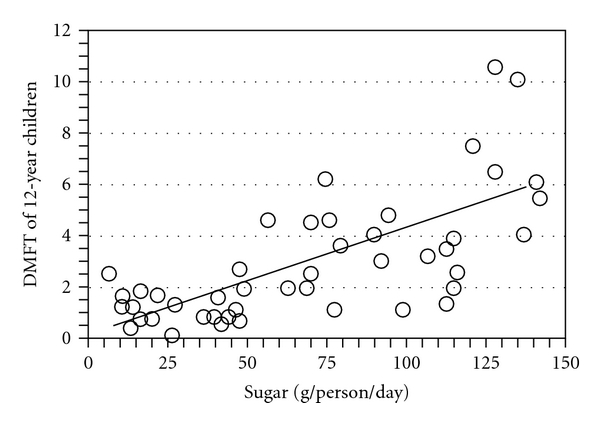
Dietary sucrose intake and dental caries severity. Each point on the graph represents a different country. Mean DMFT of the population of each country is graphed against mean sugar consumption of 12-year old children. The findings were available from World Health Organization activities in oral epidemiology and published in 1982 [10]. The graph was assembled by the author [9].

Dental caries is caused by bacterial acids within a dentally adherent biofilm (plaque) in the presence of dietary carbohydrate especially sucrose [8]. Nevertheless, the same intake of sucrose by different individuals or populations results in large disparities in caries severity. The mean number of teeth with caries in 12-year-old children from 47 different countries increases by about one decayed, missing, or filled tooth (DMFT) for every 25 g of sugar consumed daily, but there is a 50% variance in caries severity between populations, Figure 1 [9, 10]. Studies of dental caries have also established that “when individuals who have experienced dental caries…are compared with those who have remained caries-free, the most consistent difference that emerges relates to the regulation of plaque pH; caries-free subjects appear to neutralize plaque acids more effectively than those who have experienced caries” [11].

The microbiota of biofilms associated with caries is mostly gram positive and saccharolytic; the bacteria primarily metabolize glycans and excrete acids [8]. By contrast, the biofilm microbiota associated with gingivitis is mostly gram negative and asaccharolytic [12]; the bacteria primarily metabolize proteins and excrete short-chain fatty acids with ammonia, making the pH slightly alkaline [13]. Much of the gram-negative microbiota is uncommon during early childhood in Western Europe; for example, less than 5% of 5-year old Flemish children exhibit gingivitis [14]. In early childhood, therefore, saliva is a major source of base in the oral cavity. Salivary urea or free amino acids can be metabolized to provide ammonia (Section 3). Alternatively, saliva possesses basic proteins that bind to streptococci and could act as a multivalent buffer to absorb protons (Section 8). This review is written to explain why genotyping for a complex group of salivary basic proteins, the basic proline, rich protein alleles, could advance our ability to identify children who are least or most likely to develop caries.

## 2. Microbiota Variation Does Not Explain Individual Variation in Caries Severity

Studies in rodents established that a high-sucrose diet promotes the colonization of acidogenic and aciduric streptococci in the oral cavity. *Streptococcus mutans* is the most prominent of these bacteria [15] which lie within a dentally adherent biofilm along with many unrelated bacteria. Nevertheless, the association of *S. mutans* with caries is weak [16, 17] and other bacteria (*Lactobacillus* spp., *Actinomyces* spp., *Bifidobacterium* spp. and nonmutans streptococci) are increased in dental biofilms from high-caries individuals regardless of *S. mutans* colonization [18]. Indeed, a recent study of dental biofilm from young children with and without caries indicates that *S. mutans* is difficult to detect [19]. The biofilm bacteria were characterized by extracting ribosomal RNA (rRNA) and amplifying bacterial strain-specific sequences with PCR using primers to adjacent rRNA sequences which are identical in all bacteria. The obtained sequences from each individual were matched against a library of 16S rRNA from 619 bacterial species. Surprisingly, *S. mutans* was not detected unless its specific rRNA was cloned and used to detect the *S. mutans* 16S rRNA in the PCR product mixture.

Another study [20] indicates that *S. mutans* isolated from children with severe caries contains the same set of putative virulence genes as *S. mutans* from children with no caries. A third study [21] indicates that *S. mutans* DNA is detectable on teeth from only 30–40% of economically disadvantaged children irrespective of whether caries present or absent. Taken together, these new findings indicate that (1) dental caries is not caused by acids from *S. mutans* exclusively and that (2) greater acid production associated with severe early childhood caries results in mutualistic bacterial interactions [22] that differ from those in caries-free children exposed to similar socioeconomic, dietary, and fluoride environments. Dental caries apparently develops because of inefficient acid neutralization, not greater acid production.

## 3. Acquired and Intrinsic Immunity and Enamel Development in Caries

There seems little evidence for naturally acquired immunity to caries. At least 60% of new cavities develop in 20% of the population who are most affected [23] and an 8-year longitudinal study in China indicates that 94% of 3–5-year-old rural children with caries in their deciduous teeth develop caries in their permanent teeth 8 years later [24]. Differences between a cariogenic and noncariogenic microbiota must therefore be due to variations in intrinsic immunity proteins expressed from an individual's genome. Analyses of caries in monozygotic and dizygotic twins reared together and apart, or by familial linkage and association, all confirm that genetic elements are involved in determining caries severity [25]. Cariogenicity likely results from interplay between the biofilm composition, the diet, and the genetically determined host environment within in each individual's oral cavity.

One possibility is that intrinsic differences in caries development may be caused by differences in tooth enamel structure. In junctional epidermolysis bullosa, genetic mutations of laminin-5 alter enamel crystal structure as a consequence of improper basal lamina development. Junctional epidermolysis bullosa is associated with an increased risk for dental caries, but the cause may due to affected individuals following a highly refined, high-calorie diet to avoid traumatizing oral ulcers associated with this disease [26]. Changes in the organic matrix of enamel or its interface with dentin may also change the mechanical properties of enamel [27]. There are 5 major genes associated with enamel mineralization: amelogenin (AMELX), ameloblastin (AMBN), enamelin (ENAM), kallikrein (KLK4), and two tuftelin alleles (TUFT1 and TUFT 11). Children aged 3–5 with and without caries did not differ in the prevalence of polymorphisms within any of these genes, but regression analysis revealed an interaction between tuftelin and *S. mutans* that explained 26.8% of the variation in the number of decayed, missing, and filled deciduous teeth surfaces (dmfs) [28]. Different tuftelin polymorphisms may affect the susceptibility of enamel to caries in the presence of *S. mutans*. Although genetic factors affecting enamel structure influence the properties of enamel and its susceptibility to caries, they do not explain why caries-free populations neutralize acids more efficiently.

## 4. Saliva Composition and the Properties of Its Proline-Rich Proteins (PRPs)

Whole saliva is a dilute, viscous solution whose electrolytes and proteins control the microbiota and prevent tooth enamel from dissolving. Major gland secretions are obtained using devices that are held tightly by suction to the orifice of the parotid gland duct in the cheek opposite the second upper molar, or to the orifices of the submandibular and sublingual glands together beneath the tongue [29]. Human parotid saliva secretions contain small quantities of urea [30], free amino acids [11], and peptides [31] that could interact with bacterial metabolism in whole saliva to neutralize acids in the dental biofilm *in situ*. Indeed, caries-free subjects produce more ammonia from urea in their biofilm [32], although they secrete the same amount of urea in parotid and whole saliva as caries-susceptible subjects [33]. On the other hand, arginine and lysine contents are increased in the parotid saliva from caries-free individuals [11], but ammonia from arginine is increased only in whole saliva [34] and has to diffuse into the biofilm to neutralize acids [30].The greater lysine content of caries-free subjects in parotid saliva may be converted to cadaverine (a strong base) in the biofilm [35], but amounts are small, and only marginally greater than in caries-susceptible subjects [11]. Small peptides containing lysine and arginine may also be metabolized to release ammonia in the biofilm. Nevertheless, bacterial metabolism seems inadequate to explain the greater neutralization of biofilm acids in caries-free subjects (Section 1). Differences in saliva protein composition between caries-free and caries-susceptible individuals (Section 7) may provide a different and more satisfactory explanation that also accounts for intrinsic genetic differences (Section 3).

The parotid gland secretions provide about half the volume of whole saliva [29]. They contain amylase isoenzymes, proline-rich proteins (PRPs), secretory immunoglobulin A (sIgA), and small amounts of cystatins (Table 1(a)). Cystatins are peptides which inhibit cysteine proteases [36]. The submandibular/sublingual gland secretions contain salivary mucin proteins, a 50-fold greater amount of cystatins than from the parotid glands, and PRPs and sIgA in amounts similar to those from parotid glands (Table 1(b)). Amylases, cystatins, mucins, and IgA are present in whole saliva at about the concentrations expected from the combined gland secretions, but the PRPs are present at about a third of the expected amount (Table 1(c)). Small amounts of other proteins are also secreted by the salivary glands, most notably, antimicrobial histatins from the major glands [37], an antimicrobial peptide, beta defensin 1, from ductal cells of the minor salivary glands [38] and traces of proteases (Sections 6 and 8). Indeed, in order to study proteins in the major gland secretions, proteolysis must be stopped by adding protease inhibitors [39], or precipitants that denature the proteins temporarily (ammonium sulfate [40]) or permanently (trifluoroacetic acid [41]).

About 70% of the amino acids comprising the PRPs are glycine, glutamine, and proline, of which proline promotes an extended chain conformation [42]. PRPs are divided into acidic and basic families. The acidic PRPs possess a 30-amino acid N-terminal domain rich in aspartate, glutamate, and containing a few serine phosphate residues. This domain adheres strongly to recently cleaned teeth surfaces and in so doing it transmits a conformational change which exposes a previously cryptic binding site for bacteria within the nonbinding C-terminal domain [43–46]. Acidic PRPs are present in all the major salivary gland secretions but not in other gland secretions, whereas the basic PRPs are found in nasal and bronchial secretions in addition to the parotid, but not in submaxillary/sublingual secretions [47]. The basic PRPs are composed of a variable-length proline-rich domain containing arginine and lysine.

Basic PRPs do not adhere to teeth, but bind to bacteria [48–51] and polyphenols. The latter are acidic, highly toxic, protein-binding agents (also called tannins) in plant foods and drinks [41, 52]. The polymorphic basic PRPs [53] may have evolved in saliva to adsorb polyphenols and thereby increase the energy available from plants [54]. Polyphenol binding to basic PRPs also influences astringency, a sensation akin to “dry mouth” that is especially noticeable after drinking red wines [55]. Above a critical concentration, polyphenol/basic PRP complexes precipitate. Below it, the precise mixture of polyphenols determines their structure in solution [56]: compact with *π*-*π* electron-bonded phenol groups stacked above each other and pointing away from the fluid; or extended with individual phenol groups pointing into the fluid and away from each other. Compact structures bind poorly to basic PRPs, but remain in solution as colloidal complexes that promotes low astringency; extended structures interact strongly with basic PRPs, precipitate from solution, and exhibit high astringency [57]. Large basic PRPs have more affinity for polyphenols than small PRPs [54] and a lack of large PRPs may enhance astringency by promoting precipitates in which some outward pointing phenol groups remain exposed in the oral cavity. Because the intensity and duration of astringency is reduced by sucrose [58], individuals whose saliva contains mostly basic PRP fragments may prefer a cariogenic diet to reduce the astringency (Section 7).

## 5. Acidic PRP Genes and Alleles

The acidic PRP family is encoded by two genes, PRH1 and PRH2. The PRH1 locus has 3 alleles (*Db*, *Pa*, and *Pif*) that provide polymorphisms at the PRH1 locus, and 2 alleles (*Pr1* and *Pr2*) at the PRH2 locus [53]. A third allele (*Pr1 *′) is present in 16% of African-Americans in addition to the *Pr1* and *Pr2* alleles [59]. Caucasians express up to 18 combinations (polymorphisms) of these proteins in saliva (Table 2), whereas African Americans express up to 36 polymorphisms. In addition to markedly different alleles in different populations, there is linkage disequilibrium; the distribution of acidic PRP polymorphisms within a population is nonrandom [60].

**Figure 2:**
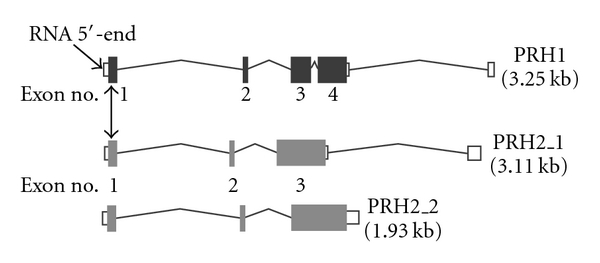
Major genes encoding the acidic proline-rich proteins. The genes are shown 5′– 3′. Dark or gray rectangles represent exons. The unfilled rectangles indicate untranslated portions of exons or whole exons which are upstream of the translational start (AUG) codon. Vertical double-pointing arrow indicates the position of the translation start codon in exon 1. Exons are numbered thereafter. The PRH1 gene shown is for alleles Pa and Pif. *Db* is encoded by the same structured gene containing an additional 63-base insert in exon 3. The two alleles of PRH2 encode separate proteins, Pr1 and Pr2. The transcripts were obtained by inserting the gene name at http://www.genecards.org/ and then selecting for the transcript diagram at ensembl.org. The transcripts are modified to read 5′–3′ and annotated.

One of the alleles encoded by PRH1 (*Db*) is unique in being 63 base pairs (21 amino acids) longer than the other 2 alleles, or either of the PRH2 alleles.  Figure 2 illustrates the intron and exon composition of the PRH1 and PRH2 alleles. When PCR was used to separate *Db* from the other alleles of PRH1 [61], the *Db* gene was found to be present in 72% of 96 African Americans and 26% of 89 Caucasians, confirming previous reports of a greater *Db* gene frequency in African Americans. Nevertheless, the gene frequency was 18% less in African Americans than the 55% gene frequency reported from determining *Db* protein in parotid saliva by gel electrophoresis [62]. This finding calls into question studies in which *Db* was detected phenotypically, such as reports that saliva-containing *Db* enhances *S. mutans* binding to saliva-coated apatite [63], or is associated with more caries in African American adults [64]. In fact, we found that cases (dmfs > 4) were significantly more common than controls (dmfs = 0) in Caucasians, and that the racial difference between cases and controls (dmfs = 0) was significant only for individuals who were *Db-negative* (*X* ^2^ = 5.6, *P* < 0.03). This finding suggests that *Db* or genes linked to *Db* in African Americans are involved in mediating less caries [61].

## 6. Basic PRP Genes and Alleles

A basic proline-rich glycoprotein and its “nonglycosylated protein core” were identified in parotid saliva more than 40 years ago [65, 66]. Neither the glycoprotein nor its “core” binds to polycations such as DEAE Sephadex and both migrate to the anode on polyacrylamide gel electrophoresis. The DEAE flow-through material (peak I) was subjected to gel filtration (Sephadex G200) and eluted as two major peaks [40], subsequently named IA and IB [67]. Peak IA contained the basic glycoprotein and peak IB contained a mixture of 9 proteins, IB-1 through IB-9, that were separated over a polyanion (SP Sephadex) with a salt gradient [40]. Similar results were obtained when proteins from a single individual were analyzed [67] and the amino acid sequences of many of these proteins were eventually reported [68, 69]. Azen and his colleagues at the University of Wisconsin independently identified genes encoding the acidic and basic PRPs on chromosome 12 [53]. His discovery enabled most of the basic PRPs to be identified as fragments of proteins encoded by four basic PRP genes [70, 71]. The basic PRPs contain 6-7% lysine plus arginine, about 20% glutamine, 20% glycine, and 40% proline [40].

**Figure 3:**
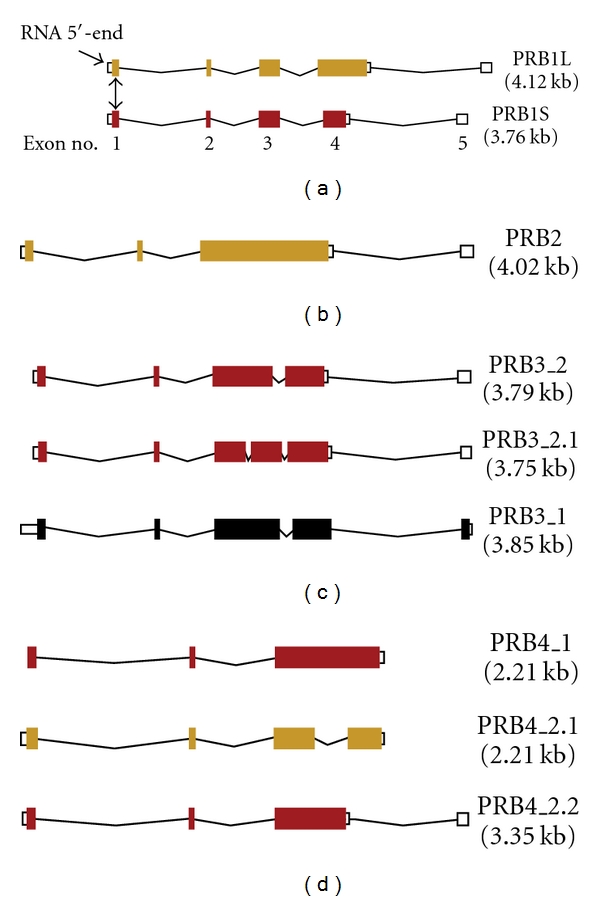
Major genes encoding the basic proline-rich proteins. Annotations and data were obtained as described in the legend to Figure 2. *Gene PRB1* (top) is polymorphic due to tandem repeats in exon 4, which encodes up to 15 sets of the repeating 20-amino acid sequence in Table 3. The alleles may be long, PRB1L (15 repeats in exon 4–ochre), or short PRB1S (9 or fewer repeats in exon 4–dark red). A medium allele (PRB1M) contains about 12 repeats (not shown). Note that exon 4 of PRB1L was modified from ensembl transcript: PRB1-001 to encode 15 repeats instead of 12. At least one allele of PRB1 is transcribed but not translated. *Gene PRB2* is similar to PRB1 in organization, size, and number of repeats, except that the repeats and most of the translated sequence occurs in exon 3. The encoded repeating amino acid sequence (Table 3) is slightly different from the repeat sequence of PRB1. *Gene PRB3* occurs as three major alleles. Unlike PRB1 and 2, there are at least one and sometimes two introns within the exon coding sequence repeat region beginning in exon 3. Exons 3 and 4 (and 5 if present) encode 10 tandem repeats of 21 amino acids. The depicted first and second alleles (Ensembl PRB1-001 and PRB3-002) encode essentially identical proteins despite the additional intron in the second, an alternate form of the long allele (PRB3L). An allele missing internal residues 158–220 (4 tandem repeats) is known as a short allele (PRB3S; not depicted). The third depicted allele is the alternative short allele (PRB3_1) which has a C-terminal deletion of 67 amino acids due to a base deletion after the first third of exon 4 (missing 3 tandem repeats) and its C-terminus consists of a sequence of 12 amino acids poorly homologous to the terminal residues of PRB3S. In some individuals, this allele is transcribed but not translated. *Gene PRB4* occurs also as three major alleles with different introns as depicted in the figure. The protein is mostly encoded in exon 3 but may differ in length. The longest allele contains 9.5 tandem repeats of 21 amino acids that are slightly different from the repeats encoded by gene PRB3. The whole protein is P10163 (UniProtKB/Swiss-Prot). The proteins reported by Esembl.org (PRB4_1 and PRB4_2.1) are identical but missing residues 113 through 154 and 164 through 184 (missing 3 repeats). The middle transcript variant has an intron within the center of exon 3, resulting in a shortened protein due to loss of its central portion (encoded residues 113 through 181; missing 6 repeats).

**Figure 4:**
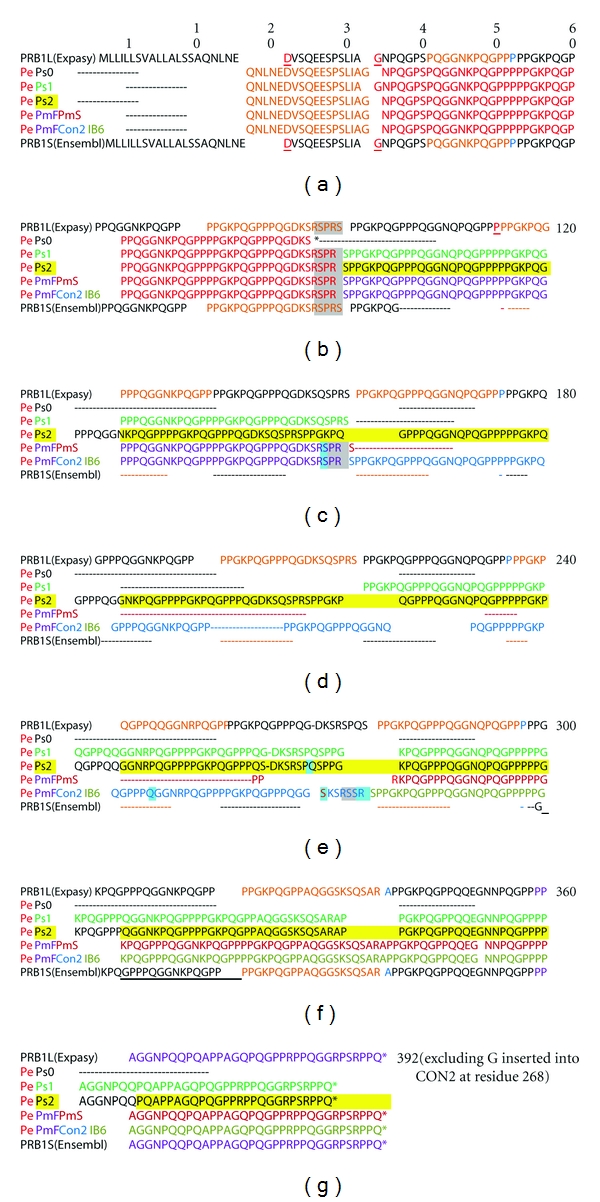
Allelic variations of PRB1 gene. *Uppermost sequence* is PRB1L from the Expasy website. The N-terminal 16 amino acids indicate the secretion signal which is cleaved within the parotid gland cells before the protein is secreted. The red amino acids (residues 22 and 34) are encoded by the joining of exons 1 and 2, and of exons 2 and 3 (Figure 3). The 15 repeats of the 20-amino acid repeating sequence (Table 3) start at residue 53 and are coded alternately orange and black. A single light blue amino acid separates some of the repeats. Residues 41–53 (orange before the first repeat) comprise the last 11 amino acids of a truncated repeat sequence. Exon 3 is connected to the variable length exon 4 (Figure 3) at residue 113 (red). *Lower sequences* show the positions of the various alleles listed in Table 4. Row 1. The N-terminal portion of Pe (orange) is encoded by exons 1 and 2. The portion of Pe encoded with other allelic proteins on exon 3 (listed with alternative names in Table 4) is shown in red. Row 2. Pe_Ps2 (red and yellow highlight) Row 3. Pe_Ps1 (red and pale green) Row 4. Pe_Ps0 (red and black) Row 5. Pe_PmF_PmS (red, purple and light blue) Row 6. Pe_PmF_Con2_IB-6 (red, purple, light blue, and pale blue). Rows 7 and 8 are translations of alleles reported by Ensembl.org, Ensembl_protein_1, and Ensembl_protein_2 (black). Both alleles likely express Pe and truncated forms of Ps1. Amino acid numbering for each allele or each furin-cleaved segment of each allele is given in Table 4. Gaps indicate deleted sequences; arrows indicate where allelic sequences differ, and underlines indicate furin cleavage sites (Section 6). The asterisk indicates the early termination of allele Ps0 in which residue 150 (R, encoded CGA) is mutated to UGA (stop). Peptide Pe is expressed, but the slightly shorter PmF-like protein is not detected [63].

**Figure 5:**
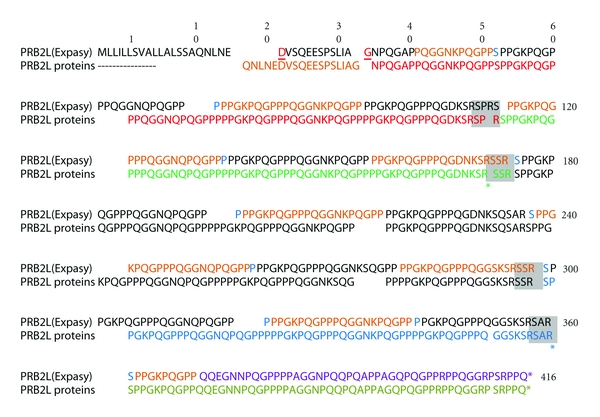
Allelic variations of PRB2 gene. *Upper sequence* is PRB2L from the Uniprot.org website. The secretion signal and two amino acids connecting the exon-encoded sequences are indicated in Figure 3 as described for Figure 4. Magenta (residues 41–51) indicates a split repeat sequence whose last 11 amino acids precede the 15 repeats of a 20-amino acid segment (Table 4). The sequences are indicated by alternating black and red colors. A green-colored amino acid (S) separates precedes the first and some later repeats. Magenta (residue 362–370) indicates the first 9 amino acids of the split sequence. *Lower sequence* indicates different allelic products. The first 17 residues of peptide IB-1 (purple) are encoded by exon2 and its downstream residues by exon 3. The products are color coded: IB-1, purple; IB-7, brown; Con1, purple; IB8c, blue; IB-4, green. Asterisk indicates R (CGA) mutated to UGA (stop) with truncation of the allele at IB-7 or IB4 [75] and is indicated by asterisks at the appropriate arginine residues. The amino acid numbering within each allele or each furin-cleaved segment of each allele is given in Table 4. Underlines indicate furin cleavage sites (Section 6).

The genes encoding the basic PRPs have greater allelic variation and more posttranslational modifications than those encoding the acidic PRPs. Diversity is due to variable numbers of repeat sequences, base changes [70, 72, 73], and posttranslational modifications such as proteolysis, phosphorylation, glycosylation, and pyroglutamate formation [74]. The variability may have evolved in saliva to enhance digestion by inactivating dietary polyphenols (Section 4).  Figure 3 illustrates the intron and exon composition of the major alleles of each PRB gene.  Table 3 lists the translated repeating sequence of each gene and Table 4 lists the names of the various genetic loci and the portions of the encoded protein that they represent. For proteins that were excised from a larger precursor protein, there is a furin-like recognition site *R*_1_ *X*_1_ *X*_2_ *R*_2_, where *X*_1_ is serine and *X*_2_ is alanine, serine, or proline. Cleavage is always at the C-terminus of R_2_, the downstream arginine residue [75]. In both PRB1 and PRB2, the codon for one of these arginine residues (CGA) may be mutated to a stop codon (UGA). The expressed alleles are truncated and many proteins are missing because of the shorter gene length (fewer repeats). The differences are illustrated in detail for PRB1 in Figure 4, and PRB2 in Figure 5. The genes encoding the basic PRPs (PRB1 through PRB4) lie together with those encoding the acidic PRPs (PRH1 and PRH2) as a cluster of about 0.7 Mbp within chromosome 12 [76]. The order (5′ to 3′) is PRB2, PRB1, PRB4, PRH2, PRB3, and PRH1, and it is therefore reasonable that PRB alleles in linkage disequilibrium with *Db* could be involved in reducing caries in African American children compared with Caucasians.

## 7. Basic PRPs and Caries

Along with urea, arginine, and lysine, one might expect the total content of basic PRPs to be increased in the parotid saliva from caries-free individuals, but this is not so [77]. On the other hand, peptides mostly derived from the basic PRPs were larger (less degraded) in the ethanol-soluble fraction of parotid saliva from each of nine individuals who were caries-free than in each of nine similarly aged individuals with severe caries [31]. Thus, the greater arginine and lysine contents of parotid secretion in caries-free individuals (Section 4) could not have been derived from basic PRP hydrolysis. Indeed, other amino acids, glutamate, histidine, methionine, and hydroxyproline, were also increased [11], suggesting that more efficient transport from blood plasma could explain the greater amino acid content.

The severe caries group had a mean DMFS of 38.4 and both groups had a mean age of about 55. The peptides from caries-free subjects were separated further into 19 peaks over a cation exchange column. Ten peaks corresponded to one of 3 basic proline-rich proteins; IB-7 and IB-4 encoded by PRB2, and IB-5 encoded by PRB4 (Table 4). Only IB-7 was more abundant in the saliva of caries-free individuals than in those with severe caries. Antiserum to the G1 glycoprotein product of PRB3 detects the products of all 4 PRB genes, and in Western blots of parotid saliva, detected Ps1 encoded by PRB1 and Con1 encoded by PRB2 in all 9 caries-free individuals, but in only 3 of 9 individuals with severe caries. No difference was detected in the frequency of PRB3 or PRB4 proteins.

Peptides from other genes were also identified in the caries-free group, IB-8b derived from the nonacidic domain of proteins encoded by PRH2 and P-B (submaxillary gland androgen-regulated protein 3A) derived from a protein encoded by gene of the same name on chromosome 4 [78]. The PRH1 and PRH2 phenotypes and the concentrations of peptides IB-8b and P-B were similar in all individuals, suggesting no association with caries. In the individuals with severe caries, the source of the many short proline-rich peptides in their saliva could not be identified because of high homology between alleles [31]. Taken together, the results of this study [31] suggest that a genetic association with caries protection may be caused by some PRB1 and PRB2 alleles being processed less than others, giving larger fragments in the parotid secretion. As noted previously (Section 4), a lack of these large PRPs may enhance the astringency of food and drink, causing an increased dietary sucrose intake that removes the astringent feeling.

More importantly, the basic PRPs in whole saliva can attach to a major adhesion antigen on the surface of *S. mutans* and other oral streptococci, cell surface protein antigen c [79]. This antigen was originally identified as the largest portion of an immunogen mixture (antigen I/II) which protects rats, hamsters, and primates from caries [80]. Much of this antigen consists of three 82-residue alanine-rich repeats (A-region) within the N-terminal third of the molecule, and three 39-residue proline-rich repeats (P-region) downstream within the central portion of the molecule [81]. The recombinant A-region was recently reported to bind PRPs, possibly by hydrophobic interactions [50]. On the other hand, the recombinant P-region binds only to the recombinant whole antigen, suggesting that it may aggregate the bacterial cells. Thus, perhaps the basic PRPs provide aggregated streptococci with a polybasic surface whose arginine and lysine residues neutralize acids from carbohydrate metabolism *in situ* (within the biofilm). *The larger the available basic PRPs, the greater will be the number of basic residues adherent to acid-producing streptococci and therefore the more efficient the acid neutralization*.

## 8. Suggested Proteomic Approaches

The greater breakdown of basic PRPs observed in adults with severe caries [31] might be due to parotid saliva containing more cysteine proteases such as cathepsin H. In the rat, cathepsin H is secreted by the pancreas, an exocrine gland closely related to the major salivary glands [82], but its presence in human salivary gland secretions appears not to have been examined. Cathepsin H cleaves *α*-amide peptide bonds [83] at the N-terminus (aminopeptidase) and internally (endopeptidase), to which actions the PRPs are highly susceptible because of their extended chain structure. Also relevant is the fact that cathepsin H does not hydrolyze imido-peptide bonds, explaining the many short proline-rich peptides in parotid saliva from the caries-susceptible group. Finally, cathepsin H and many other cysteine proteases are inhibited by cystatin C and S found in parotid gland secretions [84]. When parotid saliva is examined for cathepsin H content and its specific activity determined, the cystatin content should also be measured and compared with the specific activity. The greater the salivary cystatin content, the lower should be the cathepsin H specific activity.

Decreased cathepsin H or other protease activity in caries-free individuals could be associated with less enzyme activity, and/or more cystatin in the parotid secretion. Alternatively, large alleles such as Ps2 and Con1, respectively, encoded by PRB1 and PRB2 (Table 4), may provide larger fragments in parotid saliva. This possibility could be examined by determining the sizes of peptide fragments produced from Ps1 and Con1 after incubation with freshly collected parotid saliva from caries-free and caries-susceptible populations. Many artificial substrates are also available to measure cathepsin activity to compare with cystatin C and S content. Some fluorogenic methylcoumarylamide substrates are specific for cathepsin H whereas others are nonspecific and could be used to estimate total cysteine protease activity [85].

## 9. Suggested Genetic Approaches

Although one could examine PRP phenotypes as in the Ayad study [31], collecting parotid saliva is only possible in consenting adults. The subsequent procedures are also slow, labor-intensive, and, as discussed in Section 5, can result in misleading observations. The basic PRP fractions must be purified and the individual protein alleles separated and identified by various procedures as discussed, for example, by Amado et al. [86]. There is also a need to ensure that adult cases and controls have similar environmental factors (diet and fluoride intake) and genetic factors (see Section 5) that may confound the findings. In Rochester NY, Ayad et al. and Van Wuyckhuyse et al. [11, 31] selected their caries-free controls (DMFS = 0) by screening 4,000 individuals to identify the few life-long residents who grew up before water fluoridation and the introduction of fluoridated toothpastes, and whose teeth excluding 3rd molars were all present. An age, gender, and residence-matched group of adults who had experienced severe caries (mean DMFS = 38.4) was recruited from the same 4,000 individuals.

An easier approach would be to compare 3–5-year-old children of similar socio-economic status in the same city and exposed to the same city drinking water. A good, available group is children enrolled for at least 2 years in Head-start preschools where their diet, fluoride intake from the water supply, and use of toothpastes is controlled for most of their short lives. By contrast, such control is difficult to evaluate in adults and erroneous information can confound associations with PRP alleles. The children should be separated into those with no caries (controls, dmfs = 0), or severe caries (cases, dmfs > age in years plus 1 [87]). PRP alleles present in cases and controls can be identified using DNA collected by cheek swabbing, which is rapidly and easily performed in young children [61].

A new procedure, targeted exome capture [88], can selectively sequence the PRP protein coding regions (exons) from an individual's genome within two hours. The most expensive and time-consuming task is the synthesis of a pool of custom oligonucleotides (probes) that can be used for all subjects. The probes (attached to magnetized beads) selectively hybridize in solution to a fragmented (nebulized) sample of 5 *μ*g of genomic DNA [89], after which the beads (now including the DNA fragments of interest) are pulled down, washed to clear excess material and heated to release the attached DNA polynucleotides which are then sequenced individually. Probes are designed with a tiling algorithm around each exon of interest to ensure that no region is missed and that there are at least 10 overlapping reads. This method improves on the hybridization capture target-enrichment method by providing an excess of probes to the target region [90]. At least 20 independently obtained sequences from a single individual are compiled using software that interacts with the sequencer output to give the DNA sequence of all the exons (http://www.nimblegen.com/products/seqcap/index.html). This repetition is critical for detecting important single-base changes such as those changing arginine codons to stop codons within the PRB genes (Section 6). Ultimately, identifying all the PRP exons from enough individuals could indicate whether children with early childhood caries possess different PRP alleles compared with caries-free children of similar socioeconomic and ethnic background. If so, the most dental-caries-prone individuals could be identified at birth and better treatments developed to prevent the disease.

If some of the larger basic PRPs resist proteolysis, these alleles may explain genetic (racial) differences in caries susceptibility. Conversely, if the difference in PRP proteolysis between caries-resistant and -susceptible individuals is due to differences in endogenous (host) protease activity, the expression of basic PRP alleles will be similar in cases and controls, but the sequence of cathepsin H or cystatin C and S may differ and could be later linked to cathepsin H activity or its inhibition by cystatins. Alternatively, differences in cathepsin H expression may be identified by targeted sequence capture, of which exome capture is a specific example. Protein expression is controlled by enhancer and promoter sites upstream of the mRNA. Nucleotide polymorphisms within an upstream target region (usually less than 1-2 kb in length from the 5′ end of the mRNA) may discriminate cases from controls and could be examined for their controlling differences in cathepsin H or cystatin expression.

## 10. Conclusion

There is considerable evidence for the basic PRPs providing a genetic element in caries susceptibility. These proteins can attach to acid-producing streptococci and neutralize their acid production from carbohydrates *in situ*. There is therefore a need to determine whether the enhanced acid neutralization associated with caries protection is due to differences in basic PRP alleles secreted by the parotid gland. The primary question is whether parotid saliva from caries-susceptible individuals tends to destroy some mixtures of basic PRP alleles more than others. If no difference in basic PRP hydrolysis is found, the cause could be due to differences in activity of endoproteases, most likely cathepsin H and/or its inhibitors (cystatin C and S) in parotid saliva. The best initial approach is genetic and should utilize the new technique of targeted sequence capture. Sequencing the exons of the PRP genes along with those for cathepsin H and cystatins C and S is practicable and may ultimately provide a new method for identifying those young children who are most susceptible to severe caries.

## 11. Postscript

I conclude with the reflection that I had absolutely no idea of the potential importance of the basic PRPs when, so long ago, Pat Keller and I identified them by extending Michael Levine and Art Ellison's purification methods. 

## Figures and Tables

**Table:** (a)

Protein	*μ*g/mL min	*μ*g/mL max	^2^Fold increase	^3^% min	^4^% max
amylase	650	2600	4.0	72.1%	63.6%
cystatin	2	4	2.0	0.2%	0.1%
PRPs	230	1251	5.4	25.5%	30.6%
mucins	0	0	0.0	0.0%	0.0%
sIgA	20	230	11.5	2.2%	5.6%

Total	902	4085			

**Table:** (b)

Protein	*μ*g/mL min	*μ*g/mL max	Fold increase	% min	% max
amylase	0	0	0.0	0.0%	0.0%
cystatin	92	280	3.0	19.0%	12.6%
PRPs	270	1335	4.9	55.9%	59.8%
mucins	80	560	7.0	16.6%	25.1%
sIgA	41	56	1.4	8.5%	2.5%

Total	483	2231			

**Table:** (c)

Protein	*μ*g/mL min	*μ*g/mL max	Fold increase	% min	% max
amylase	380	500	1.3	46.4%	23.8%
cystatin	240	280	1.2	29.3%	13.3%
PRPs	90	180	2.0	11.0%	8.6%
mucins	90	700	7.8	11.0%	33.3%
sIgA	19	439	23.1	2.3%	20.9%

Total	819	2099			

^1^Data rearranged from of [91, Table 1].

^2^Fold increase is the minimal (min) to maximal (max) concentration (*μ*g/mL), giving the minimal concentration a value of 1.

^3^% min-% of min total content

^4^% max-% of max total content.

**Table 2:** All possible combinations of expressed PRH1 alleles. The three proteins encoded by the PRH1 locus are on the left two columns and the two proteins encoded by the PRH2 locus in Caucasians on the right two columns. Because this locus is expressed from both parental genes (12A and 12B), there are six possible protein (allelic) combinations of *Pa*, *Pif*, and *Db* and three possible combinations of *Pr1* and *Pr2*. This gives a total of 18 possible combinations (polymorphisms) among individuals. A single Caucasian individual has one of the six combinations encoded by the PRH1 locus paired with one of the three combinations encoded by the PRH2 locus [9].

PRH1 locus^1^		PRH2 locus^1^	
Chromosome 12A Protein	Chromosome 12B Protein	Chromosome 12A Protein	Chromosome 12B Protein
*Db*	*Db*	PRP-1	PRP-1
*Db*	*Pa*	PRP-1	PRP-2
*Db*	*Pif*	PRP-2	PRP-2
*Pa*	*Pa*		
*Pa*	*Pif*		
*Pif*	*Pif*		

^1^Total = 6 PRH1 allelic combinations X 3 PRH2 combinations = 18.

**Table 3:** List of PRB protein repeats.

PRB1	
9, 12, or 15 repeats of a 20-amino acid sequence: P-P-G-K-P-Q-G-P-P-[PAQ]-Q-[GE]-[GD]-[NKS]-[KSQRN]-[PRQS]-[QS] [GPS]-[PQAR]-[PSR]	

PRB2	
9, 12, or 15 repeats of a 20-amino acid sequence: P-P-G-K-P-Q-G-P-P-P-Q-G-[GD]-[NKS]-[KSQ]-[PRS]-[QRS] [GPS]-[PSAR]-[PSR]	

PRB3	
6, 7, or 10 repeats of a 21-amino acid sequence: [RH]-P-G-K-P-[EQ]-G-[PQS]-P-[PS]-Q-[GE]-G-N-[QK]-[SP]-[QR]-[GR]-P-P-P	

PRB4	
3.5, 6.5, or 9.5 repeats of a 21-amino acid sequence: AA tandem repeats of K-P-[EQ]-[GR]-[PR]-[PR]-P-Q-G-G-N-Q-[PS]-[QH]-[RG]-[PT]-P-P-[PH]-P-G with the last repeat being truncated at residue 11–N)	

**Table 4:** Basic PRP genes and products^1^.

Locus	Name of encoded protein^2^	Partial	Residues^3^
PRB1:	Basic salivary proline-rich protein 1		1–392
		Pe (II-2)	17–91
		Ps2	92–392
		IB-9 (PmF)	91–152
		Ps1 (deletion)^4^	92–153; 213–392
		Con2 (deletion)^4^	152–194; 214–274
		IB-6 (PmS, P-I)^5^	275–392

PRB2:	Basic salivary proline-rich protein 2		1–416
	(Con1 glycoprotein)	IB-1	17–112
		IB-7 (P-G)	113–171^6^ or 174^7^
		Con1	175–299
		IB-8C (P-F)	299–359
		IB-4 (P-H)	361–416

PRB3:	Basic salivary proline-rich protein 3		1–309
	(Parotid salivary glycoprotein G1)	G1	17–309

PRB4:	Basic salivary proline-rich protein 4		1–310
	(Parotid o protein)	Protein N1	17–39
	(Salivary proline-rich protein II-1)	Glycosylated Pr A	40–177
		IB-5 (P-D)	241–310

^1^From Azen et al. [70, Table 1] and from Ayad et al. [31, Table 3] updated from the Protein Knowledgebase website (UniProt.org) which was accessed from GeneCards or Epasy websites.

^2^There is extensive polymorphism (see text). Many proteins encoded by PRB1 and PRB2 [60], Ps1, Ps2, PmS PmF, Pe, Con2 are not included in the Protein Knowledgebase, or (for Con1) the composition is improperly indicated as an alternative name for the whole gene product, which it is not (see Figure 5).

^3^First set of numbers for each gene indicates the full length encoded polypeptide of each gene. The numbers beneath indicate the fragments of the polypeptide that are commonly found in parotid saliva secretions. The N-terminal 16 amino acids comprise a secretion signal which is removed prior to secretion (Figure 4).

^4^Deletion of repeating sequences 154–212 or 195–213

^5^Sequence is identical to IB-8c plus IB-4. IB-4 (P-H) is not normally released from IB-6 because residues 333–336 are QSAR (Figure 4). IB-4 (P-H) is normally derived from PRB2 where the last 4 C-terminal amino acids of IB-8c (356–360) are RSAR (see text, Section 6). At this time, Uniprot.org appears to have incorrectly listed P-H as produced by PRB1, but IB4 correctly from PRB2. P-H is an alternative name for IB4 and would only be produced from PRB1 if the codon for Q (residue 333) is mutated to R, an uncommon A to G mutation, or an even less common double mutation.

^6,7^Termination codon in PRB2S, or cleavage in PRB2M (see legend to Figure 5).
